# Skin-brain axis in Alzheimer's disease - Pathologic, diagnostic, and therapeutic implications: A Hypothetical Review

**DOI:** 10.14336/AD.2024.0406

**Published:** 2024-04-06

**Authors:** Hyeon soo Kim, Haram Jung, Yong Ho Park, Su-Hak Heo, Sujin Kim, Minho Moon

**Affiliations:** ^1^Department of Biochemistry, College of Medicine, Konyang University, Daejeon 35365, Korea.; ^2^Department of Medicinal Bioscience, Konkuk University (Glocal Campus), Chungcheongbuk-do 27478, Korea.; ^3^Research Institute for Dementia Science, Konyang University, Daejeon 35365, Korea.

**Keywords:** Alzheimer's disease, skin-brain axis, skin disease, neurodegeneration

## Abstract

The dynamic interaction between the brain and the skin is termed the 'skin-brain axis.' Changes in the skin not only reflect conditions in the brain but also exert direct and indirect effects on the brain. Interestingly, the connection between the skin and brain is crucial for understanding aging and neurodegenerative diseases. Several studies have shown an association between Alzheimer's disease (AD) and various skin disorders, such as psoriasis, bullous pemphigoid, and skin cancer. Previous studies have shown a significantly increased risk of new-onset AD in patients with psoriasis. In contrast, skin cancer may reduce the risk of developing AD. Accumulating evidence suggests an interaction between skin disease and AD; however, AD-associated pathological changes mediated by the skin-brain axis are not yet clearly defined. While some studies have reported on the diagnostic implications of the skin-brain axis in AD, few have discussed its potential therapeutic applications. In this review, we address the pathological changes mediated by the skin-brain axis in AD. Furthermore, we summarize (1) the diagnostic implications elucidated through the role of the skin-brain axis in AD and (2) the therapeutic implications for AD based on the skin-brain axis. Our review suggests that a potential therapeutic approach targeting the skin-brain axis will enable significant advances in the treatment of AD.

## 1. Introduction

The skin is the first organ in the body to respond to progressive changes during aging [1]. It is the largest organ in the body and undergoes both extrinsic and intrinsic aging processes. The skin acts as a window for age-related pathophysiological changes in the internal organs through progressive immunologic dysregulation, increased thinning of the epidermal barrier, and glycosylation of dermal extracellular matrix proteins [2]. In particular, a previous study proposed the concept of a 'skin-brain axis' to describe the dynamic interaction between the brain and skin [3]. Skin alterations not only reflect the state of the brain but can also directly or indirectly influence the condition of the brain. For example, increased systemic pro-inflammatory cytokines due to trauma or skin diseases can affect the regulation of blood-brain barrier (BBB) permeability and immunomodulation in the brain [4]. In addition, damaged skin-induced immunological changes contribute to alterations in neurotransmission and neuropsychiatric and behavioral function [4]. Conversely, long-term changes in brain conditions, including chronic stress and major depressive disorder, can modulate the immune profile of the skin and exacerbate skin diseases, such as psoriasis, through the hypothalamic-pituitary-adrenal axis and hormonal regulation [5].

Furthermore, neurodegenerative disease-associated proteins, such as amyloid beta (Aβ) and α-synuclein, accumulate in the skin as well as in the brain [6]. Remarkably, the skin and brain originate from the same ectoderm and share the molecular pathways involved in aging and the onset of neurodegenerative diseases [7]. Consequently, the skin can serve as a non-invasive window for observing and understanding various neuropathologies and various neurodegenerative diseases [8].

Alzheimer’s disease (AD) is the most prevalent neurodegenerative disease among the elderly [9]. AD is characterized by the accumulation of Aβ and hyperphosphorylated tau [9]. The deposition of Aβ and hyperphosphorylated tau in the AD brain leads to irreversible cognitive impairment via the induction of neuroinflammation, synaptic loss, and neuronal cell death [10]. In particular, both neurodegenerative alterations in the brain and complex physiological communication between the brain and the periphery are factors that contribute to AD progression [11, 12]. Previous studies have reported a relationship between AD and skin diseases [6, 13, 14]. One study found that patients with AD had a 2.6-fold increased risk of developing bullous pemphigoid (BP) [14]. Thus, exploring the connection between the skin-brain axis and AD pathology is a novel contribution to the study of the pathomechanisms of AD [15].

Accumulating evidence suggests that the brain and skin not only mirror each other's condition but also actively participate in maintaining homeostasis and impact health through their intricate and reciprocal interactions. The complex interactions between the brain and skin during aging and neurodegenerative processes underlie the importance of the skin as a crucial indicator and contributor to neurodegeneration. However, the importance of the skin-brain axis in the diagnosis, progression, and treatment of AD has not been clearly established. In this review, we elucidate (1) the pathological alterations mediated by the skin-brain axis in AD, (2) the diagnostic implications of the skin-brain axis in AD, and (3) the therapeutic implications of AD elucidated through the skin-brain axis. Moreover, we aimed to provide a more detailed understanding of the pathophysiological and molecular alterations observed in skin disorders related to AD.

**Figure 1.**
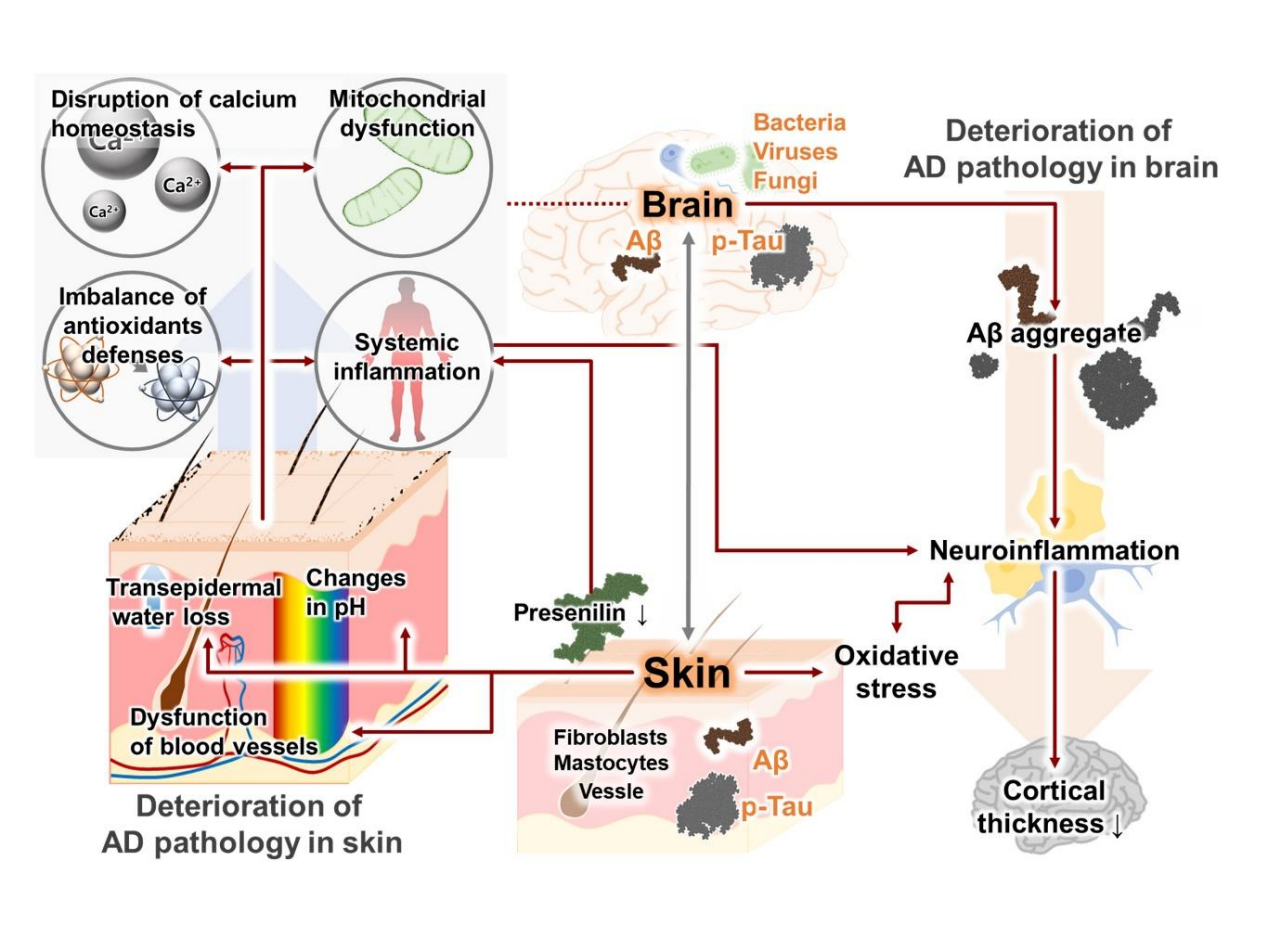
**Overview of the association between AD-related pathologic features in the skin and brain**. The solid line indicates known connections, and the dotted line indicates hypothesized connections.

## 2. Skin-brain axis in AD

### 2.1. AD-related pathological changes mediated by the skin-brain axis

Deposition of Aβ plaque in the brain is the hallmark pathology of AD [16]. Interestingly, the accumulation of Aβ also occurs in the skin, particularly in fibroblasts [17]. Recent research has suggested that antioxidant defenses are lower in skin fibroblasts from patients with familial AD than in those from controls. Furthermore, skin fibroblasts from AD patients exhibit a variety of dysfunctions, such as abnormally activated bradykinin receptor signaling, altered cholesterol ester metabolism, disrupted calcium homeostasis, and impaired mitochondrial function (Table 1) [18-21]. Moreover, accumulated Aβ alters the function of blood vessels in the skin, similar to the changes that are observed in the brains of patients with AD [22, 23]. In addition, decreased expression of presenilin-1, which is involved in degrading the amyloid precursor protein (APP), has been associated with seborrheic keratosis and inflammatory skin disease in patients with AD [24]. Interestingly, Aβ and tau have also been shown to accumulate in skin mastocytes [25, 26]. Accumulated Aβ and tau have been reported to trigger epidermal keratinization-induced inflammation, pH changes, and transepidermal water loss in AD patients [27, 28]. These studies indicate the importance of understanding the skin-brain axis, as changes related to Aβ and tau in the skin are associated with the pathological features of the AD brain (Fig. 1).

**Table 1:** Pathological evidence of the skin-brain interconnection in animal models of AD and patients with AD.

Subjects	Age	Main findings	Ref.
**Presenilin knock-down mice**	6-month-old	Decreased expression of presenilin 1 leads to the development of seborrheic keratoses and inflammatory skin conditions	[24]
**AD patients**	-	Aβ and tau accumulated in the skin mastocytes of patients with AD. Aβ and tau accumulated in the mastocytes induced epidermal keratinization changes in pH, and transepidermal water loss in the skin of patients with AD.	[28]
**Skin fibroblasts derived from AD patients**	56.7-years-old	Skin fibroblasts derived from AD patients exhibited lower antioxidant defenses compared to those from normal subjects	[18]
**Skin fibroblasts derived from AD patients**	63.2-years-old	Skin fibroblasts derived from AD patients exhibit selective phosphorylation of tau protein on Ser residues.	[19]
**Skin fibroblasts derived from AD patients**	72.0-years-old	Skin fibroblasts from AD patients exhibited the dysregulation of cholesterol homeostasis.	[21]

Abbreviation: Aβ, Amyloid-β; Alzheimer's disease, AD; potential of hydrogen, pH; serine, Ser.

Surprisingly, several studies have revealed a potential correlation between AD and skin diseases such as psoriasis, BP, and skin cancer. Patients with psoriasis tend to develop mild cognitive impairment (MCI) earlier than healthy individuals, which may be associated with a higher risk of AD onset [29, 30]. Considering that early is more strongly associated with an increased risk of progression to AD than late MCI [31], psoriasis may have important implications as a risk factor for AD. Furthermore, patients with psoriasis have decreased cortical thickness in the parahippocampal gyrus, superior temporal gyrus, and superior frontal gyrus [30, 32]. Previous studies reported that morphological changes, including reduced cortical thickness, could be potent indicators of the MCI to AD transition [33]. A genome-wide association study (GWAS) to identify genetic links between AD and psoriasis found shared genetic factors in both diseases and suggested that psoriasis, which induces a chronic inflammatory response, may affect neuroinflammation and increase the risk of AD [34]. In addition, the association between psoriasis and AD is much stronger in middle-aged patients (40-64 years) than in older patients [35]. Patients with psoriasis treated with anti-inflammatory drugs have a lower risk of developing AD than controls without psoriasis. Futhermore, systemic administration of anti-inflammatory drugs may reduce the development of AD by reducing neuroinflammation as well as systemic inflammation. Thus, considering that patients with psoriasis have a greater tendency to develop MCI and show AD-related pathologies, such as reduced cortical thickness in certain brain regions, psoriasis and its pathologies can be considered potential risk factors contributing to the development of AD.

Blistered skin disease, an autoimmune disorder that causes large and dense blistering of the skin of older adults, has been associated with neurological diagnoses including AD, stroke, and Parkinson's disease (PD) [36]. Notably, patients with blisters have significantly lower cognitive scores than healthy controls, suggesting that patients with blisters are at a higher risk of developing cognitive impairment [37]. Surprisingly, BP autoantigens are expressed not only in the skin but also in the brain [38]. Recent studies have reported that patients with AD are more likely to be positive for BP autoantibodies than healthy controls [38, 39]. Considering the increased risk of BP development in patients with AD [39], it is believed that BP contributes to AD-related pathophysiology via autoantibodies that act on both the skin and the brain.

Interestingly, some studies have suggested that infectious diseases causing skin lesions may also be associated with AD. A number of recent studies have presented evidence supporitng the ‘infection hypothesis’, that infection by bacteria, viruses, and fungi contributes to the pathogenesis and progression of AD [40]. Although the mechanisms by which fungal skin infections contribute to AD pathology are not fully understood, one study showed that fungal skin infections may contribute to AD progression and cognitive decline [41]. For instance, infection with *Malassezia* spp. induces skin inflammation and reduces skin barrier integrity [42]. Chronic skin deficits caused by *Malassezia* spp. infections provide an opportunity for the skin microbiome to enter the bloodstream and brain [41]. A postmortem study showed that *Malassezia globosa* and *Malassezia restrica* were distributed in the brains of patients with AD [43]. *Malassezia* spp. not only increase the levels of interleukin (IL)-17 and IL-23, but also upregulate the expression of toll-like receptor 4, activate T helper (Th)1/Th17 cells, and induce microglial phagocytic dysfunction by enhancing the expression of caspase recruitment domain family member 9 [41]. Therefore, skin infections caused by *Malassezia* spp. may contribute to Aβ deposition, neuroinflammation, and cognitive impairment. In addition, skin infections with *Cladosporium* spp. can contribute to AD pathology by enhancing neuroinflammation in the AD brain [41]. Next-generation sequencing studies on the postmortem brains of patients with AD support the influence of skin disease-causing fungi on AD pathogenesis [43]. Moreover, some viruses that cause skin lesions may also contribute to AD pathology and cognitive impairment. Although it remains controversial whether *Herpesviridae* directly promotes or accelerates AD pathology [44-46], some studies suggest that *Herpesviridae* infection may be a potential risk factor for AD [47, 48]. In particular, varicella zoster virus (VZV) and herpes simplex virus type (HSV)-1, which cause varicella and oral herpes, respectively [49], have been proposed as risk factors for the progression of AD pathology [50, 51]. A recent study has shown that the superinfection of VZV on quiescent HSV-1-infected human-induced neuronal stem cells reactivates HSV-1 to contribute to Aβ and tau pathology [52]. In addition, VZV infection and VZV gB peptide have been reported to increase production and self-aggregation of Aβ in human primary astrocytes [53]. Several studies have suggested that infection by HSV-1 could exacerbate Aβ and tau pathology in the brain [48]. Similar to HSV-1, HSV-2 may also deteriorate Aβ pathology via alteration of APP processing [54]. Importantly, VZV and HSV not only infect epithelial cells and induce lesions in the skin but can also infect neurons and contribute to AD-associated neuropathology. Exploring and characterizing other skin-infecting viruses that may induce AD-associated neurodegeneration via the skin-brain axis is an interesting topic for future studies.

Previous studies have shown a significantly decreased risk for AD in patients with skin cancer [55-57]. Indeed, cancer and AD involve opposing processes: unrestrained proliferation in cancer and apoptosis in AD [58]. In other words, cancer activates signaling pathways that promote cell survival, whereas AD promotes signaling pathways that lead to increased cell death. Therefore, the signaling pathways involved in cell survival that are upregulated in skin cancer may contribute to the mitigation of neurodegeneration in AD. In skin cancer, the enzyme Pin1, which plays a role in protein folding and cell cycle control [59], is up-regulated, whereas the level of Pin1 is decreased in the brain of patients with AD [55, 60]. Particularly, Pin1 can regulate AD-related proteins including tau and APP [61, 62]. In AD, reduced activity of Pin1 decreases isomerization of APP and tau protein, leading to the development of Aβ- and tau-related cell death and pathology. In skin cancer, the expression of Pin1 is increased, resulting in the prevention of Aβ- and tau-related pathology in the brain. Moreover, the overexpression of Pin1 may attenuate neurodegeneration by activating the Wnt/beta-catenin signaling pathway [63]. Many studies investigating the relationship between skin cancer and AD have suggested that alterations in DNA methylation and the activity of the p53 tumor suppressor gene may contribute to AD pathology [57, 64, 65]. These results suggest that the pathways promoting cell survival in skin cancer may contribute to the reduced neurodegeneration observed in patients with AD. In summary, various skin diseases and causative pathogens may influence AD-related pathology.

### 2.2. Skin-brain axis as a diagnosis strategy in AD

The diagnostic relevance of the skin-brain axis has been emphasized in a broad spectrum of neurodegenerative diseases. In particular, the skin reflects the pathological progression of neurodegeneration. Interestingly, skin biopsy has been suggested as an emerging new diagnostic method for PD, Lewy body dementia (LBD), and AD [28]. Measuring the seeding activity of α-synuclein aggregates in skin tissue has received much attention as a diagnostic technique for PD [66]. Surprisingly, the analysis of an autopsied abdominal skin sample using real-time quiver induction conversion succeeded in diagnosing PD with 95% sensitivity and 100% specificity [66]. In addition, measurements of phosphorylated α-synuclein deposited on autonomic skin nerve fibers clearly discriminated patients with LBD from patients with non-synucleinopathy dementia, as well as healthy controls [67]. These studies clearly show that protein-related alterations in the skin are effective diagnostic markers for neurodegenerative diseases. Several recent studies have attempted to develop an AD diagnostic method using skin biopsies [8, 28]. Transcriptomic analysis of patients with AD showed that some genes, including those in keratinocytes, fibroblasts, and endothelial cells, are differentially expressed in AD [68]. Frequent transcriptional alterations were observed in genes encoding the low-density lipoprotein receptor (LDLR), interferon-stimulated gene 15 (ISG15), galactin-3-binding protein (GAL3BP), and copine 1 (CPNE1) in the skin cells of patients with AD [68]. LDLR, which is involved in lipid and energy metabolism, is altered in the AD brain [69]. LDLR has been proposed as an important factor contributing to the progression of Aβ and tau pathology by interacting with apolipoprotein E [69]. Moreover, ISG15, a key regulator of interferon-related immunity, shows different expression levels in the parahippocampal gyrus of patients with MCI and AD compared to healthy controls [70]. Furthermore, GAL3BP may modulate AD pathology by blocking the β-cleavage of APP and suppressing the activity of galactin-3, which is abnormally regulated in AD [71]. A previous study suggested that GAL3BP levels in serum or cerebrospinal fluid (CSF) could be potential biomarkers for AD [71]. Collectively, the investigation of transcriptional changes in genes associated with AD, such as *CPNE1*, *ISG15*, *LDLR*, and *GAL3BP*, whose expressions are altered in the skin cells of AD patients, may contribute to the development of AD diagnostic tools and methods using the skin. The oral mucosa can also serve as a potential biomarker for diagnosing AD as it originates from ectodermal tissues similar to those of the skin and brain [72, 73]. Accumulated evidence suggests that buccal cells and fibroblast cells in AD patients display specific alterations that could be valuable in the AD diagnosis. Buccal cells from AD patients show significant differences compared to age-matched controls in both cytosolic components and DNA [74-76]. Furthermore, studies involving buccal cytome assay on the buccal mucosa of patients with AD reported altered tissue kinetics compared to age-matched controls [76]. One study that examined telomeres in buccal cells using three-dimensional imaging revealed differences in telomere morphology between buccal cells derived from age-matched individuals and those derived from patients with AD. Furthermore, significant changes in the telomere architecture of buccal cells have been observed, depending on the stage of AD progression [77]. One study combining ATR-FTIR spectrometry and analytical algorithms demonstrated significant differences in the composition of biomolecules present in buccal cells between controls and AD patients [74]. In another study using skin punch biopsies, the use of phosphorylated ERK1/2 in fibroblasts as a biomarker was reported to potentially provide higher accuracy, sensitivity, and specificity in the diagnosis of AD than diagnosis by autopsy or clinical methods [78]. One study reported a significant increase in Aβ levels of oral mucosal cells through multi-parameter analysis [79]. Although relatively few studies have focused on oral mucosal epithelial cells for AD diagnosis, further research on the potential of using the oral mucosa could significantly contribute to the diagnosis of AD. Nevertheless, the reduction in the ratio of basal, karyorrhectic, and condensed chromatin cells in the buccal mucosa of AD patients suggests that AD-related physiological changes may affect the kinetics of various cells or tissues constituting the buccal mucosa. Taken together, these results suggest that AD-related changes along the skin-brain axis may be a valid approach for the diagnosis of AD.

Currently, amyloid positron emission tomography (PET) is the most effective method for diagnosing AD, and has been shown to be capable to classify healthy individuals and AD patients with high sensitivity and specificity (Table 2) [80]. Unfortunately, it has some disadvantages owing to its low accessibility and high cost [81]. Therefore, several studies have been conducted to develop simpler, safer, and more economical technologies for AD diagnosis. In particular, measuring the changes in the blood or CSF biomarkers for the AD diagnosis is considered a promising approach [82]. The sensitivity and specificity of AD diagnosis using CSF are very high [83, 84]. In particular, one study demonstrated that CSF biomarkers provide high sensitivity (81%) and specificity [81%] for diagnosing AD, with positive and negative predictive values of 87% and 72 %, respectively [84]. Moreover, recent studies on AD diagnosis using blood have shown that blood-based diagnostic tools exhibit sensitivities ranging from 83% to 96% and specificities ranging from 76% to 100% [85]. However, invasive methods have problems, such as concerns regarding side effects such as CSF leakage and infection at the sample collection site, as well as issues related to pain, patient reluctance, and uncooperativeness during the test [86]. The establishment of skin-based AD diagnostics may provide a relatively non-invasive, low-risk, inexpensive, and convenient means of sample collection and AD diagnosis. Therefore, the use of AD-related changes along the skin-brain axis may be a potential approach for a more beneficial and safe diagnostic strategy for neurodegenerative diseases.

**Table 2:** Comparison of sensitivity and specificity of diagnostic methods for AD.

Method	Biomarker	Number of participants [AD patients/controls]	Sensitivity	Specificity	Ref.
**Amyloid PET** **(Florbetaben)**	Aβ	81/69	80%	91%	[87]
**Tau PET**	Tau	6/21	100%	86%	[88]
CSF	Aβ_42/40_ ratio	55/34	51%	82%	[89]
	**Aβ_42_**	22/35	100%	80%	[90]
	**Aβ_42/40_ ratio**	22/35	95.2%	88.4%	
	**Aβ_42_**	48/107	94%	72%	[91]
	**Aβ_42/40_ ratio**	48/107	92%	79%	
	**Aβ_42_**	100/50	98.0	74.0	[92]
	**Aβ_42/40_ ratio**	100/50	85.7	78.0	
	**Aβ_42/38_ ratio**	100/50	81.6	82.0	
Blood	Plasma Aβ_42_	- (Meta-analysis)	88%	81%	[85]
	**Plasma Aβ oligomer**	- (Meta-analysis)	80%	88%	
	**Plasma tau**	- (Meta-analysis)	90%	87%	
**Skin biopsy (Buccal cell)** **/ Buccal cytome assay**	Ratio of buccal cell and micronuclei	54/66	82%	97%	[76]
**Skin biopsy (Buccal cell)** **/ ATR-FTIR spectroscopy**	Composition of biomolecules in buccal cell	17/12	76%	100%	[74]
**Skin biopsy (Buccal cell)** **/ Laser scanning cytometry+ Oil Red O staining**	DNA content and neutral lipid content	13/26	62%	100%	[75]
**Skin biopsy (Skin fibroblast)** **/ Morphometric imaging**	Morphology of skin fibroblast	25/21	100%	100%	[93]
**Skin biopsy (Skin fibroblast)** **/ Transketolase assay**	Transketolase in skin fibroblast	38/12	69.4%	100%	[94]

Abbreviation: Alzheimer's disease, AD; Aβ, Amyloid-β; PET, Positron Emission Tomography; CSF, cerebrospinal fluid; DNA, Deoxyribonucleic acid.

### 2.3. Modulation of the skin-brain axis as a therapeutic target in AD

The skin is involved in the peripheral nervous systems (PNS), autonomic nervous systems (ANS), and central nervous systems (CNS). Interestingly, in the context of skin homeostasis and disease management, substantial evidence suggests that the cutaneous PNS plays a crucial role in the skin-brain axis [95]. Several studies have demonstrated that the vagus nerve, which is distributed in the skin, can modulate the neuroendocrine and neuroimmune systems. In particular, transcutaneous vagus nerve stimulation (tVNS), an intervention that involves electrical stimulation of the vagus nerve through the skin, has provided important insights into the skin-brain axis. tVNS promotes anti-inflammatory signaling of the vagus nerve via the α7nAchR/ nuclear factor kappa-light-chain-enhancer of activated B cells (NF-κB) signaling pathway [96]. In addition, tVNS induces microglial switching to an anti-inflammatory phenotype, and alleviates the excessive secretion of pro-inflammatory cytokines through the P2X7R/NLRP3/Caspase-1 signaling pathway in APP/PS1 mice [97, 98]. Surprisingly, recent studies are attempting to treat neurological disorders through tVNS [98]. tVNS improved locus coeruleus activity and memory performance in healthy older adults [99]. Moreover, tVNS, which stimulates the auricular branch of the vagus nerve, significantly restores cognitive dysfunction in individuals with MCI [100]. Recent literature and clinical trials (NCT number: NCT02363504) have suggested that tVNS can be applied in patients with AD to effectively ameliorate cognitive impairment [99, 101]. These findings suggest that skin stimulation via tVNS can mitigate cognitive dysfunction by modulating neuroendocrine and neuroimmune systems through the vagus nerve and enhancing brain connectivity.

**Table 3:** Therapeutic interventions targeting the skin-brain axis for AD-related pathology in AD animal models and AD patients.

Therapeutic interventions		Subjects	Age	Main findings	Ref. / Trial number
**Dermatological drugs**	**Etanercept** /psoriasis, psoriatic arthritis	Aβ-injected Swiss mice	6-weeks-old	Treatment with etanercept attenuated cognitive impairment mediated by Aβ.	[102]
		AD patients	76.7-years-old	Treatment with etanercept attenuated cognitive impairment.	[103]
		AD patients	81-years-old	Treatment with etanercept attenuated cognitive impairment.	[104]
		AD patients	72.4-years-old	Peripherally administered etanercept showed a trend toward improvement in ADL and cognitive impairment, although the difference was not statistically significant.	[105] NCT01068353
	**Infliximab** /psoriasis, rashes, skin lesions, ulcers, hives, and swollen face or lips	APP/PS1 mice	12-months-old	Administration of infliximab reduced amyloid plaques, tau phosphorylation, and level of TNF-α.	[106]
		Aβ oligomer-injected ICR mice	6-weeks-old	Administration of infliximab ameliorated the impairment of AD-associated recognition memory and blocked Aβ toxicity.	[107]
		AD patients	57-years-old	Treatment of infliximab attenuated cognitive impairment and increased the concentration of Aβ_1-42_ and p-tau in CSF and blood.	[108]
	**Thalidomide** /erythema nodosum leprosum	Aβ-injected ICR mice	-	Treatment of thalidomide attenuated cognitive impairment caused by Aβ.	[109]
	**Rapamycin** /atopic dermatitis, psoriasis, skin aging	3xTg mice	6- and 12-month-old	Rapamycin administration rescued AD-related early learning and memory deficits and reduced Aβ and tau pathology.	[110]
	**Minocycline** /pimples, red bumps	J20 mice	8- and 12-months-old	Minocycline reduced Aβ burden and restored AD-associated cognitive dysfunction.	[111]
		Aβ-injected rat	10-weeks-old	Minocycline decreased neuronal cell death and mitigated learning and memory impairment caused by Aβ.	[112]
		Thy1-APP mice	3-months-old	Minocycline reduced microgliosis and Aβ burden and inhibited BACE-1 activity.	[113]
		Mu p75-SAP-injected mice	3-months-old	Minocycline reduced microgliosis and astrogliosis. Minocycline attenuated cognitive impairment.	[114]
		Tg-SwDI mice	12-months- old	Minocycline reduced microgliosis and restored spatial learning memory impairment.	[115]
	**Celastrol** /psoriasis. atopic dermatitis	APP/PS1 mice	6-months-old	Minocycline reduced microgliosis and Aβ burden.	[116]
	**Bexarotene** /skin cancer	APP/PS1 mice	6-months-old	Bexarotene attenuated cognitive impairment. Bexarotene reduced Aβ burden and rescued cortical network activity.	[117]
**tVNS**		APP/PS1 mice	6- and 12-months-old	tVNS induced a morphological transition of microglia from neurodestructive to neuroprotective phenotype.	[97]
		APP/PS1 mice	6-months-old	tVNS improved spatial memory and learning tVNS decreased neuroinflammatory response, Aβ deposits, and neurodegeneration. tVNS inhibits the hippocampal P2X7R/NLRP3/Caspase-1 signaling.	[98]
		AD patients	60~85-years-old	Modulating the locus coeruleus function through tVNS	NCT02363504
**Dermal patch**		AD patients	55~95-years-old	Rivastigmine dermal patch showed numerical improvement in CASI and MMSE scores.	[118]
		AD patients	-	Rivastigmine dermal patch significantly reduced blood esterase levels, specifically BuChE.	[119, 120]
		AD patients	50~85-years- old	Rivastigmine dermal patch demonstrated improvement or stability in the ADAS-Cog scores.	[121]
		AD patients	-	Rivastigmine dermal patch demonstrated restoration of cognitive dysfunction.	[122]

Abbreviation: Alzheimer's disease, AD; Aβ, Amyloid-β; ADL, Activities of Daily Living; BACE-1, beta-site amyloid precursor protein cleaving enzyme 1; CSF, cerebrospinal fluid; TNF-α, tumor necrosis factor-α; tVNS, Transcutaneous vagus nerve stimulation; CASI, cognitive assessment screening instrument; MMSE, mini-mental state examination; BuChE, butyrylcholinesterase; ADAS-Cog, Alzheimer's Disease Assessment Scale-Cognitive Subscale.

Moreover, a better understanding of the close relationship between AD neuropathology and increased systemic inflammatory responses or microbial invasion in skin diseases will provide opportunities to develop new therapeutic interventions for AD. Numerous studies support the beneficial effects of dermatological drugs on AD by modulating inflammatory and infectious responses, reducing Aβ and tau accumulation, and inhibiting neurodegeneration (Table 3). A recent study reported that patients with psoriasis treated with etanercept, or infliximab had a reduced risk of AD [123]. Etanercept or infliximab are known to be effective in the treatment of psoriasis, in which T cells and dendritic cells activated by tumor necrosis factor (TNF) accumulate and produce cytokines such as TNF-α and IL-23 [124]. Surprisingly, TNF-α, a key pro-inflammatory factor, is also known to play an important role in the pathogenesis of AD and to exacerbate Aβ and tau pathology [125]. It has been showed that intraventricular injection of infliximab into APP/PS1 mice reduced the levels of TNF-α, the accumulation of Aβ, and the phosphorylation of tau in the brain [106]. Similarly, Aβ oligomer-injected ICR mice exhibited recovery of memory impairment upon intraventricular administration of infliximab [107]. Moreover, thalidomide, used to treat erythema lepromatosis, restored cognitive dysfunction in Aβ-injected ICR mice [109]. In a study of patients with AD, perispinal administration of etanercept resulted in significant recovery of cognitive decline [103, 104], and intrathecal administration of infliximab resulted in the restoration of cognitive dysfunction [108]. Furthermore, etanercept has been shown to slow cognitive decline and improve activities of daily living in patients with AD in phase 1 and 2 clinical trials (NCT number: NCT01068353) [105]. Thus, there is both preclinical and clinical evidence to suggest that exploiting the mechanisms of skin disease drugs, such as administration of TNF-α inhibitors, may be an effective strategy for treating AD. However, since the TNF-α inhibitors used to treat skin diseases, such as etanercept and infliximab, do not cross the BBB, it is difficult for them to act directly in the brain. Nevertheless, the TNF-α inhibitor etanercept, is known to regulate the immune response within the CNS and AD pathology, despite its poor BBB permeability [126, 127]. Recent studies have revealed that etanercept alleviates neuroinflammation through the inhibition of c-Jun N-terminal kinase and NF-kB pathways and exerts neuroprotective effects in AD [128]. In addition, etanercept reduced microgliosis, tau phosphorylation, and neuronal loss in the brains of PS19 transgenic mice, a model of tauopathy [129]. Interestingly, TNF-α inhibitors, whether or not they penetrate the BBB, have been shown to suppress tau-related neuroinflammation and neurodegeneration [129]. This result suggests that etanercept may alleviate AD-related pathology by reducing systemic TNF-α levels, rather than by directly affecting glial cells in the brain [129]. Similarly, TNF-α inhibitors have been reported to attenuate Aβ accumulation independent of BBB penetration [127]. Interestingly, BBB-penetrating TNF-α inhibitors have been shown to regulate AD-associated pathology independently of inflammation modulation associated with Iba-1 or CD11b reduction [127]. These results indicate that TNF-α inhibitors, independent of BBB permeability, may alleviate the pathophysiology of AD by reducing systemic inflammation and skin infections.

Notably, it is well known that the fungi that cause skin disorders destroy the epithelial barrier of the skin and enter the brain through systemic infection [41]. *Candida* spp., *Malassezia* spp., *Cladosporium* spp., and *Alternaria* spp. are among the most common fungi found in the brain of patients with AD. Fungi are believed to contribute to AD pathogenesis in the brain through amyloidogenic processing and gliosis [130, 131]. These four fungi may contribute to inflammatory responses and degeneration of both the brain and skin. Interestingly, a recent study investigated the utility of miconazole, an antifungal skin ointment widely used to inhibit fungal cell membrane synthesis, in the treatment of lipopolysaccharide (LPS)-induced AD in animal models [132]. Miconazole ameliorates memory decline by inhibiting the expression of inducible nitric oxide synthase and alleviates neuroinflammation by downregulating the level of the NF-κB protein in LPS-treated AD mice [132]. Although miconazole was not used to treat fungal infections in an AD-induced mouse model, these experiments suggest that targeting changes in the skin-brain axis can ameliorate the AD pathology. These results suggest a potential dual role for miconazole in the treatment of AD pathology and fungal infections.

Since most treatments for skin diseases target inflammatory responses, it is reasonable to assume that they also exert therapeutic effects on AD. In AD, Aβ overproduction and tau hyperphosphorylation lead to the formation of Aβ/tau aggregates, which are neurotoxic and induce immune responses in microglia. Microglial activation results in the release of pro-inflammatory cytokines and neurotoxic factors, such as TNF-α and IL-1β, which not only amplify the inflammatory response but also trigger neuronal cell death. When neurons are destroyed, microglial activity is further enhanced by the release of microglial activating factors, such as laminin and neuromelanin. This self-perpetuating cycle contributes to the escalation of inflammatory responses in AD [133]. Breaking this cycle by administering dermatological drugs that inhibit inflammation may potentially slow the pathological deterioration and contribute to the treatment of AD. It has been suggested that drug repositioning for AD could potentially be facilitated by the conjugation of BBB transporters to various TNF-α inhibitors used for skin diseases or by chemical modification of drugs to increase BBB permeability. For example, a chemically modified BBB-permeable version of thalidomide has been found to exert neuroprotective effects [134]. Rapamycin and minocycline, known for their high BBB permeability, have been reported to improve BBB dysfunction in other disease models [135, 136]. In addition, Celastrol, which penetrates the BBB, may directly mitigate AD pathology by activating transcription factor EB (TFEB)-mediated autophagy in the brain. Furthermore, bexarotene nano-encapsulated to facilitate drug delivery effectively alleviated Aβ pathology [137].

The presence of amyloid in the skin could plausibly trigger protein aggregation, including Aβ and tau, in the brain, influencing AD pathology. The established skin-brain connection offers a promising avenue for investigating drug delivery via skin administration as a safe and simple approach for treating AD. Recent research has actively focused on the development of therapeutic agents using transdermal drug delivery systems such as dermal patches. Dermal patches are medical devices that deliver drugs through the skin, facilitating gradual release which facilitates absorption into the bloodstream. This method offers the advantage of efficiently delivering the required drug doses to patients with AD, particularly to those who may experience gastrointestinal side effects or face challenges with drug administration owing to swallowing difficulties and memory impairment. In particular, rivastigmine, a cholinesterase inhibitor, is an agent for which a transdermal delivery system has been developed, and is currently prescribed and used for patients with mild to severe AD. The rivastigmine dermal patches have shown several beneficial effects, including reduced blood esterase levels, particularly BuChE, and increased food intake in patients with AD [119, 120]. Furthermore, some studies have demonstrated that using a rivastigmine transdermal patch in patients with AD can help to restore cognitive dysfunction [118, 122]. Notably, a transdermal patch of rivastigmine was found to have equivalent efficacy to rivastigmine capsules [122], suggesting that the transdermal delivery of rivastigmine through the patch may offer therapeutic benefits for patients with AD. Collectively, recognizing the importance of the interconnection in the skin-brain axis in AD and utilizing skin disease drugs may provide new approaches for the treatment of AD.

## 3. Limitation and perspective

As AD is a complex disease characterized by multiple pathologies, it is important to identify any potential associations with various factors such as Aβ, tau, neuroinflammation, and mitochondrial dysfunction to clarify the role of the skin-brain axis in AD. However, most existing studies on the skin-brain connection have focused on the correlation between skin conditions and the incidence of AD, or skin conditions and AD-related genes, rather than adequately addressing the direct association of skin conditions with AD pathology. Previous studies made it difficult to integrate and standardize the function of the skin-brain axis in AD. Large-scale prospective studies with longer follow-up periods are therefore required to clarify the complex relationships between AD and various skin diseases. Clinical studies in the medical domain aimed at exploring interventions targeting the relationship between the skin and the brain have potential to offer valuable insights into potential therapeutic and diagnostic approaches for AD. Additional non-clinical studies are also needed to better understand the skin-brain axis. To explore the mechanisms underlying the relationship between skin disease and the progression of AD, examining the changes in AD-related pathology, including Aβ pathology, hyperphosphorylated tau, neuroinflammation, neurodegeneration, and mitochondria dysfunction, by triggering skin cancer or skin disease in AD animal models, are still need. Moreover, it is essential to investigate changes in both the skin and brain from the early developmental stages to the onset of AD. Studies examining changes in skin disease models injected with Aβ or tau may suggest a relationship between skin disease and the development of AD. Furthermore, collaboration among dermatologists, neurologists, and neuroscientists is essential to ensure a comprehensive and multidisciplinary approach to understanding the pathological, diagnostic, and therapeutic role of skin-brain relationships in AD.

**Figure 2.**
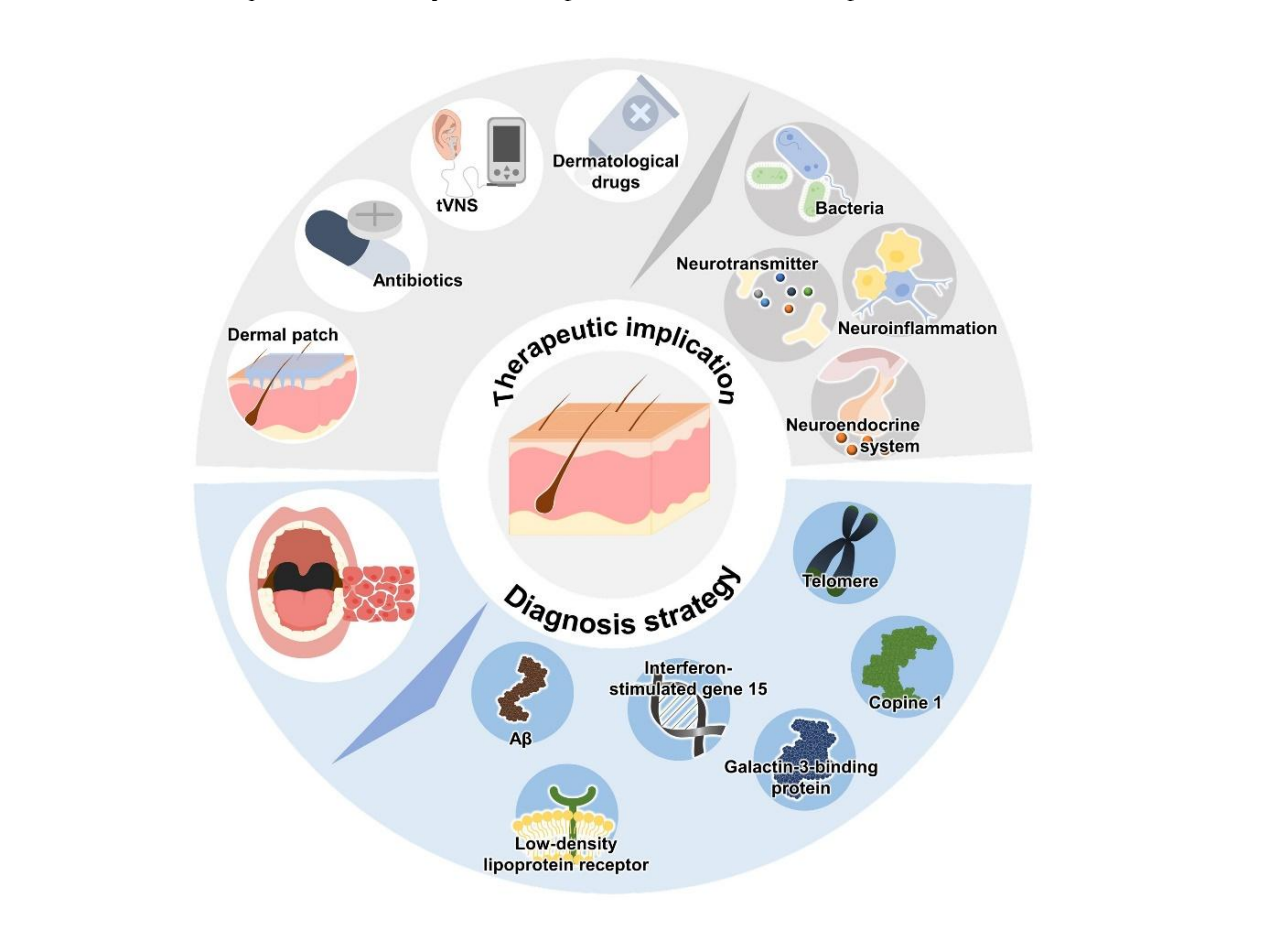
Strategies for the diagnosis and treatment of AD *via* targeting of the skin-brain axis.

In terms of the development of AD diagnostic technologies, the most significant barriers and challenges faced by existing AD diagnostic technologies include: (1) distinguishing between MCI and healthy individuals, (2) distinguishing between MCI and AD, (3) early prediction of MCI progression to AD, (4) invasive methods (5) high cost, and (6) low accessibility for participants. In this study, we propose that skin biopsy can help to address the issue of the high cost of AD diagnostic technology and offers a more non-invasive approach compared to existing methods. Unfortunately, it has not yet been established whether the diagnostic method of AD through skin biopsy can also be used to distinguish between patients with MCI and those with AD with high sensitivity and specificity. Future research should validate whether skin biopsy can differentiate between MCI and AD, as well as predict the progression from MCI to AD In addition, although skin biopsy has advantages over traditional diagnostic methods in the diagnosis of AD in terms of invasiveness, cost, and convenience, more evidence is required before it can be commercially applied. Furthermore, more detailed studies on the mechanisms by which skin cells exhibit specific changes in AD are needed to establish reliable biomarkers for AD. Further mechanistic studies are also needed to compare the accuracy, sensitivity, and specificity of newly discovered skin AD biomarkers.

## 4. Conclusions

In this review, we elucidated the pathophysiological relationship between the skin and brain in AD. In addition, we describe the possibility of applying skin-based diagnostic and therapeutic approaches that reflect the role of the skin-brain axis in AD (Fig. 2). Although the skin and brain are usually considered distinct organs, they share a common embryological origin and pathophysiological characteristics such as inflammation. Skin changes may serve as indicators of brain changes that reflect the onset and progression of AD and vice versa. Therefore, it is important to identify the AD-related pathological changes mediated by the skin-brain axis, and utilizing the skin-brain axis may be helpful in the diagnosis and treatment of AD. Moreover, the repurposing of drugs for the treatment of AD-related skin diseases may be an interesting avenue for the development of novel therapeutic strategies for AD.

## References

[1] Nikolakis G, Makrantonaki E, Zouboulis CC (2013). Skin mirrors human aging. Horm Mol Biol Clin Investig, 16:13-28. 10.1515/hmbci-2013-0018 25436743

[2] MakrantonakiE, SchonknechtP, HossiniAM, KaiserE, KatsouliMM, AdjayeJ, et al. (2010). Skin and brain age together: The role of hormones in the ageing process. Exp Gerontol, 45:801-813.20719245 10.1016/j.exger.2010.08.005

[3] ArckP, HandjiskiB, HagenE, PincusM, BruenahlC, BienenstockJ, et al. (2010). Is there a 'gut-brain-skin axis'? Exp Dermatol, 19:401-405.20113345 10.1111/j.1600-0625.2009.01060.x

[4] HadianY, FregosoD, NguyenC, BagoodMD, DahleSE, GareauMG, et al. (2020). Microbiome-skin-brain axis: A novel paradigm for cutaneous wounds. Wound Repair Regen, 28:282-292.32034844 10.1111/wrr.12800

[5] WeigleinA, GaffalE, AlbrechtA (2022). Probing the Skin-Brain Axis: New Vistas Using Mouse Models. Int J Mol Sci, 23.35806489 10.3390/ijms23137484PMC9267936

[6] AkermanSC, HossainS, ShoboA, ZhongY, JourdainR, HancockMA, et al. (2019). Neurodegenerative Disease-Related Proteins within the Epidermal Layer of the Human Skin. J Alzheimers Dis, 69:463-478.31006686 10.3233/JAD-181191

[7] JamesonC, BoultonKA, SiloveN, NananR, GuastellaAJ (2023). Ectodermal origins of the skin-brain axis: a novel model for the developing brain, inflammation, and neurodevelopmental conditions. Mol Psychiatry, 28:108-117.36284159 10.1038/s41380-022-01829-8PMC9812765

[8] ZouboulisCC, MakrantonakiE, HossiniAM (2021). Skin Mirrors Brain: A Chance for Alzheimer's Disease Research. Adv Exp Med Biol, 1339:371-380.35023127 10.1007/978-3-030-78787-5_45

[9] KnopmanDS, AmievaH, PetersenRC, ChetelatG, HoltzmanDM, HymanBT, et al. (2021). Alzheimer disease. Nat Rev Dis Primers, 7:33.33986301 10.1038/s41572-021-00269-yPMC8574196

[10] RajmohanR, ReddyPH (2017). Amyloid-Beta and Phosphorylated Tau Accumulations Cause Abnormalities at Synapses of Alzheimer's disease Neurons. J Alzheimers Dis, 57:975-999.27567878 10.3233/JAD-160612PMC5793225

[11] TrushinaE (2019). Alzheimer's disease mechanisms in peripheral cells: Promises and challenges. Alzheimers Dement (N Y), 5:652-660.31720366 10.1016/j.trci.2019.06.008PMC6838468

[12] KimHS, KimS, ShinSJ, ParkYH, NamY, KimCW, et al. (2021). Gram-negative bacteria and their lipopolysaccharides in Alzheimer's disease: pathologic roles and therapeutic implications. Transl Neurodegener, 10:49.34876226 10.1186/s40035-021-00273-yPMC8650380

[13] ClosAL, KayedR, Lasagna-ReevesCA (2012). Association of skin with the pathogenesis and treatment of neurodegenerative amyloidosis. Front Neurol, 3:5.22319507 10.3389/fneur.2012.00005PMC3262151

[14] ForstiAK, JokelainenJ, AnsakorpiH, SeppanenA, MajamaaK, TimonenM, et al. (2016). Psychiatric and neurological disorders are associated with bullous pemphigoid - a nationwide Finnish Care Register study. Sci Rep, 6:37125.27845416 10.1038/srep37125PMC5109264

[15] EiserAR, FulopT (2023). Alzheimer's Disease Is a Multi-Organ Disorder: It May Already Be Preventable. J Alzheimers Dis, 91:1277-1281.36617785 10.3233/JAD-221078

[16] MurphyMP, LeVineH3rd (2010). Alzheimer's disease and the amyloid-beta peptide. J Alzheimers Dis, 19:311-323.20061647 10.3233/JAD-2010-1221PMC2813509

[17] Soininen H, Syrjanen S, Heinonen O, Neittaanmaki H, Miettinen R, Paljarvi L, et al. (1992). Amyloid beta-protein deposition in skin of patients with dementia. Lancet, 339:245. 10.1016/0140-6736(92)90046-6 1346198

[18] CecchiC, FiorilloC, SorbiS, LatorracaS, NacmiasB, BagnoliS, et al. (2002). Oxidative stress and reduced antioxidant defenses in peripheral cells from familial Alzheimer's patients. Free Radic Biol Med, 33:1372-1379.12419469 10.1016/s0891-5849(02)01049-3

[19] JongYJ, FordSR, SeehraK, MalaveVB, BaenzigerNL (2003). Alzheimer's disease skin fibroblasts selectively express a bradykinin signaling pathway mediating tau protein Ser phosphorylation. FASEB J, 17:2319-2321.14563691 10.1096/fj.02-1147fje

[20] OkadaA, UrakamiK, TakahashiK, OhnoK, SatoK, EndoH (1994). Expression of amyloid beta-protein precursor mRNAs in cultured skin fibroblasts taken from patients with dementia of the Alzheimer type. Dementia, 5:55-56.8156089 10.1159/000106697

[21] PaniA, DessiS, DiazG, La CollaP, AbeteC, MulasC, et al. (2009). Altered cholesterol ester cycle in skin fibroblasts from patients with Alzheimer's disease. J Alzheimers Dis, 18:829-841.19749436 10.3233/JAD-2009-1193

[22] KalmanJ, SzakacsR, TorokT, RozsaZ, BarzoP, RudasL, et al. (2002). Decreased cutaneous vasodilatation to isometric handgrip exercise in Alzheimer's disease. Int J Geriatr Psychiatry, 17:371-374.11994892 10.1002/gps.609

[23] KhalilZ, LoGiudiceD, KhodrB, MaruffP, MastersC (2007). Impaired peripheral endothelial microvascular responsiveness in Alzheimer's disease. J Alzheimers Dis, 11:25-32.17361032 10.3233/jad-2007-11106

[24] TournoyJ, BossuytX, SnellinxA, RegentM, GarmynM, SerneelsL, et al. (2004). Partial loss of presenilins causes seborrheic keratosis and autoimmune disease in mice. Hum Mol Genet, 13:1321-1331.15128703 10.1093/hmg/ddh151

[25] Kvetnoi IM, Kvetnaia TV, Riadnova I, Fursov BB, Ernandes-Jago H, Blesa JR (2003). [Expression of beta-amyloid and tau-protein in mastocytes in Alzheimer disease]. Arkh Patol, 65:36-39. 12669611

[26] SchremlS, KaiserE, LandthalerM, SzeimiesRM, BabilasP (2010). Amyloid in skin and brain: what's the link? Exp Dermatol, 19:953-957.20807234 10.1111/j.1600-0625.2010.01166.x

[27] KlostermeierS, LiA, HouHX, GreenU, LennerzJK (2023). Exploring the Skin Brain Link: Biomarkers in the Skin with Implications for Aging Research and Alzheimer's Disease Diagnostics. Int J Mol Sci, 24.37686115 10.3390/ijms241713309PMC10487444

[28] WuCY, HoCY, YangYH (2023). Developing Biomarkers for the Skin: Biomarkers for the Diagnosis and Prediction of Treatment Outcomes of Alzheimer's Disease. Int J Mol Sci, 24.37239825 10.3390/ijms24108478PMC10218562

[29] EspinosaA, AlegretM, ValeroS, Vinyes-JunqueG, HernandezI, MauleonA, et al. (2013). A longitudinal follow-up of 550 mild cognitive impairment patients: evidence for large conversion to dementia rates and detection of major risk factors involved. J Alzheimers Dis, 34:769-780.23271318 10.3233/JAD-122002

[30] GisondiP, SalaF, AlessandriniF, AvesaniV, ZoccatelliG, BeltramelloA, et al. (2014). Mild cognitive impairment in patients with moderate to severe chronic plaque psoriasis. Dermatology, 228:78-85.24434720 10.1159/000357220

[31] JessenF, WolfsgruberS, WieseB, BickelH, MoschE, KaduszkiewiczH, et al. (2014). AD dementia risk in late MCI, in early MCI, and in subjective memory impairment. Alzheimers Dement, 10:76-83.23375567 10.1016/j.jalz.2012.09.017

[32] AlmkvistO, AxelmanK, BasunH, JensenM, ViitanenM, WahlundLO, et al. (2003). Clinical findings in nondemented mutation carriers predisposed to Alzheimer's disease: a model of mild cognitive impairment. Acta Neurol Scand Suppl, 179:77-82.12603253 10.1034/j.1600-0404.107.s179.11.x

[33] GomarJJ, Bobes-BascaranMT, Conejero-GoldbergC, DaviesP, GoldbergTE, Alzheimer's Disease Neuroimaging I (2011). Utility of combinations of biomarkers, cognitive markers, and risk factors to predict conversion from mild cognitive impairment to Alzheimer disease in patients in the Alzheimer's disease neuroimaging initiative. Arch Gen Psychiatry, 68:961-969.21893661 10.1001/archgenpsychiatry.2011.96

[34] YokoyamaJS, WangY, SchorkAJ, ThompsonWK, KarchCM, CruchagaC, et al. (2016). Association Between Genetic Traits for Immune-Mediated Diseases and Alzheimer Disease. JAMA Neurol, 73:691-697.27088644 10.1001/jamaneurol.2016.0150PMC4905783

[35] KimM, ParkHE, LeeSH, HanK, LeeJH (2020). Increased risk of Alzheimer's disease in patients with psoriasis: a nationwide population-based cohort study. Sci Rep, 10:6454.32296117 10.1038/s41598-020-63550-2PMC7160134

[36] TaghipourK, ChiCC, VincentA, GrovesRW, VenningV, WojnarowskaF (2010). The association of bullous pemphigoid with cerebrovascular disease and dementia: a case-control study. Arch Dermatol, 146:1251-1254.21079062 10.1001/archdermatol.2010.322

[37] ZhaoW, WangY, MaoX, PayneAS, FengS, LiW, et al. (2020). Detection of underlying dementia in bullous pemphigoid patients using cognitive evaluation tests: a multicenter case-control study. Ann Transl Med, 8:1397.33313142 10.21037/atm-20-1319PMC7723532

[38] KokkonenN, HerukkaSK, HuilajaL, KokkiM, KoivistoAM, HartikainenP, et al. (2017). Increased Levels of the Bullous Pemphigoid BP180 Autoantibody Are Associated with More Severe Dementia in Alzheimer's Disease. J Invest Dermatol, 137:71-76.27650606 10.1016/j.jid.2016.09.010

[39] WangYN, HammersCM, MaoX, JinHZ, YuanJ, LiL (2021). Analysis of the autoimmune response against BP180 in patients with Alzheimer's disease. Ann Transl Med, 9:107.33569409 10.21037/atm-20-5343PMC7867912

[40] SeaksCE, WilcockDM (2020). Infectious hypothesis of Alzheimer disease. PLoS Pathog, 16:e1008596.33180879 10.1371/journal.ppat.1008596PMC7660461

[41] PhunaZX, MadhavanP (2022). A closer look at the mycobiome in Alzheimer's disease: Fungal species, pathogenesis and transmission. Eur J Neurosci, 55:1291-1321.35048439 10.1111/ejn.15599

[42] SparberF, De GregorioC, SteckholzerS, FerreiraFM, DolowschiakT, RuchtiF, et al. (2019). The Skin Commensal Yeast Malassezia Triggers a Type 17 Response that Coordinates Anti-fungal Immunity and Exacerbates Skin Inflammation. Cell Host Microbe, 25:389-403 e386.30870621 10.1016/j.chom.2019.02.002

[43] AlonsoR, PisaD, Fernandez-FernandezAM, CarrascoL (2018). Infection of Fungi and Bacteria in Brain Tissue From Elderly Persons and Patients With Alzheimer's Disease. Front Aging Neurosci, 10:159.29881346 10.3389/fnagi.2018.00159PMC5976758

[44] BocharovaOV, FisherA, PanditNP, MolesworthK, MychkoO, ScottAJ, et al. (2023). Abeta plaques do not protect against HSV-1 infection in a mouse model of familial Alzheimer's disease, and HSV-1 does not induce Abeta pathology in a model of late onset Alzheimer's disease. Brain Pathol, 33:e13116.36064300 10.1111/bpa.13116PMC9836376

[45] MurphyMJ, FaniL, IkramMK, GhanbariM, IkramMA (2021). Herpes simplex virus 1 and the risk of dementia: a population-based study. Sci Rep, 11:8691.33888766 10.1038/s41598-021-87963-9PMC8062537

[46] SchnierC, JanbekJ, LatheR, HaasJ (2022). Reduced dementia incidence after varicella zoster vaccination in Wales 2013-2020. Alzheimers Dement (N Y), 8:e12293.35434253 10.1002/trc2.12293PMC9006884

[47] WainbergM, LuquezT, KoelleDM, ReadheadB, JohnstonC, DarvasM, et al. (2021). The viral hypothesis: how herpesviruses may contribute to Alzheimer's disease. Mol Psychiatry, 26:5476-5480.33972690 10.1038/s41380-021-01138-6PMC8758477

[48] ProttoV, MarcocciME, MitevaMT, PiacentiniR, Li PumaDD, GrassiC, et al. (2022). Role of HSV-1 in Alzheimer's disease pathogenesis: A challenge for novel preventive/therapeutic strategies. Curr Opin Pharmacol, 63:102200.35276497 10.1016/j.coph.2022.102200

[49] KennedyPG, RovnakJ, BadaniH, CohrsRJ (2015). A comparison of herpes simplex virus type 1 and varicella-zoster virus latency and reactivation. J Gen Virol, 96:1581-1602.25794504 10.1099/vir.0.000128PMC4635449

[50] BernsteinHG, KeilhoffG, DobrowolnyH, SteinerJ (2020). Binding varicella zoster virus: an underestimated facet of insulin-degrading enzyme s implication for Alzheimer s disease pathology? Eur Arch Psychiatry Clin Neurosci, 270:495-496.30806771 10.1007/s00406-019-00995-1

[51] ItzhakiRF (2018). Corroboration of a Major Role for Herpes Simplex Virus Type 1 in Alzheimer's Disease. Front Aging Neurosci, 10:324.30405395 10.3389/fnagi.2018.00324PMC6202583

[52] CairnsDM, ItzhakiRF, KaplanDL (2022). Potential Involvement of Varicella Zoster Virus in Alzheimer's Disease via Reactivation of Quiescent Herpes Simplex Virus Type 1. J Alzheimers Dis, 88:1189-1200.35754275 10.3233/JAD-220287

[53] BubakAN, ComoCN, CoughlanCM, JohnsonNR, HassellJE, MescherT, et al. (2020). Varicella-Zoster Virus Infection of Primary Human Spinal Astrocytes Produces Intracellular Amylin, Amyloid-beta, and an Amyloidogenic Extracellular Environment. J Infect Dis, 221:1088-1097.31665341 10.1093/infdis/jiz560PMC7075411

[54] ShipleySJ, ParkinET, ItzhakiRF, DobsonCB (2005). Herpes simplex virus interferes with amyloid precursor protein processing. BMC Microbiol, 5:48.16109164 10.1186/1471-2180-5-48PMC1198230

[55] IblerE, TranG, OrrellKA, SerranoL, MajewskiS, SableKA, et al. (2018). Inverse association for diagnosis of Alzheimer's disease subsequent to both melanoma and non-melanoma skin cancers in a large, urban, single-centre, Midwestern US patient population. J Eur Acad Dermatol Venereol, 32:1893-1896.29573497 10.1111/jdv.14952PMC6153078

[56] SchmidtSA, OrdingAG, Horvath-PuhoE, SorensenHT, HendersonVW (2017). Non-melanoma skin cancer and risk of Alzheimer's disease and all-cause dementia. PLoS One, 12:e0171527.28225789 10.1371/journal.pone.0171527PMC5321271

[57] WhiteRS, LiptonRB, HallCB, SteinermanJR (2013). Nonmelanoma skin cancer is associated with reduced Alzheimer disease risk. Neurology, 80:1966-1972.23677746 10.1212/WNL.0b013e3182941990PMC3716346

[58] GanguliM (2015). Cancer and Dementia: It's Complicated. Alzheimer Dis Assoc Disord, 29:177-182.25710132 10.1097/WAD.0000000000000086PMC4437917

[59] MinSH, ZhouXZ, LuKP (2016). The role of Pin1 in the development and treatment of cancer. Arch Pharm Res, 39:1609-1620.27572155 10.1007/s12272-016-0821-x

[60] MalterJS (2023). Pin1 and Alzheimer's disease. Transl Res, 254:24-33.36162703 10.1016/j.trsl.2022.09.003PMC10111655

[61] BalastikM, LimJ, PastorinoL, LuKP (2007). Pin1 in Alzheimer's disease: multiple substrates, one regulatory mechanism? Biochim Biophys Acta, 1772:422-429.17317113 10.1016/j.bbadis.2007.01.006PMC1868500

[62] DriverJA, ZhouXZ, LuKP (2014). Regulation of protein conformation by Pin1 offers novel disease mechanisms and therapeutic approaches in Alzheimer's disease. Discov Med, 17:93-99.24534472 PMC4076490

[63] NakamuraK, KosugiI, LeeDY, HafnerA, SinclairDA, RyoA, et al. (2012). Prolyl isomerase Pin1 regulates neuronal differentiation via beta-catenin. Mol Cell Biol, 32:2966-2978.22645310 10.1128/MCB.05688-11PMC3434519

[64] BehrensMI, LendonC, RoeCM (2009). A common biological mechanism in cancer and Alzheimer's disease? Curr Alzheimer Res, 6:196-204.19519301 10.2174/156720509788486608PMC2810550

[65] TremolizzoL, Rodriguez-MenendezV, BrighinaL, FerrareseC (2006). Is the inverse association between Alzheimer's disease and cancer the result of a different propensity to methylate DNA? Med Hypotheses, 66:1251-1252.16459025 10.1016/j.mehy.2005.12.022

[66] Wang Z, Becker K, Donadio V, Siedlak S, Yuan J, Rezaee M, et al. (2020). Skin alpha-Synuclein Aggregation Seeding Activity as a Novel Biomarker for Parkinson Disease. JAMA Neurol. 10.1001/jamaneurol.2020.3311 PMC7522783 32986090

[67] DonadioV, IncensiA, RizzoG, CapellariS, PantieriR, Stanzani MaseratiM, et al. (2017). A new potential biomarker for dementia with Lewy bodies: Skin nerve alpha-synuclein deposits. Neurology, 89:318-326.28667178 10.1212/WNL.0000000000004146

[68] MukhamedyarovMA, RizvanovAA, YakupovEZ, ZefirovAL, KiyasovAP, ReisHJ, et al. (2016). Transcriptional Analysis of Blood Lymphocytes and Skin Fibroblasts, Keratinocytes, and Endothelial Cells as a Potential Biomarker for Alzheimer's Disease. J Alzheimers Dis, 54:1373-1383.27589530 10.3233/JAD-160457PMC5260811

[69] LeeJ, ChoiJ, WongGW, WolfgangMJ (2016). Neurometabolic roles of ApoE and Ldl-R in mouse brain. J Bioenerg Biomembr, 48:13-21.26686234 10.1007/s10863-015-9636-6PMC4733629

[70] RoyER, WangB, WanYW, ChiuG, ColeA, YinZ, et al. (2020). Type I interferon response drives neuroinflammation and synapse loss in Alzheimer disease. J Clin Invest, 130:1912-1930.31917687 10.1172/JCI133737PMC7108898

[71] SekiT, KanagawaM, KobayashiK, KowaH, YahataN, MaruyamaK, et al. (2020). Galectin 3-binding protein suppresses amyloid-beta production by modulating beta-cleavage of amyloid precursor protein. J Biol Chem, 295:3678-3691.31996371 10.1074/jbc.RA119.008703PMC7076203

[72] ThedaC, HwangSH, CzajkoA, LokeYJ, LeongP, CraigJM (2018). Quantitation of the cellular content of saliva and buccal swab samples. Sci Rep, 8:6944.29720614 10.1038/s41598-018-25311-0PMC5932057

[73] FrancoisM, LeifertW, MartinsR, ThomasP, FenechM (2014). Biomarkers of Alzheimer's disease risk in peripheral tissues; focus on buccal cells. Curr Alzheimer Res, 11:519-531.24938500 10.2174/1567205011666140618103827PMC4166904

[74] Paraskevaidi M, Karim S, Santos M, Lima K, Crean S The use of ATR-FTIR spectroscopy for the diagnosis of Alzheimer’s disease using oral buccal cells. Applied Spectroscopy Reviews:1-15.

[75] FrancoisM, LeifertW, HeckerJ, FauntJ, MartinsR, ThomasP, et al. (2014). Altered cytological parameters in buccal cells from individuals with mild cognitive impairment and Alzheimer's disease. Cytometry A, 85:698-708.24616437 10.1002/cyto.a.22453

[76] ThomasP, HeckerJ, FauntJ, FenechM (2007). Buccal micronucleus cytome biomarkers may be associated with Alzheimer's disease. Mutagenesis, 22:371-379.17709794 10.1093/mutage/gem029

[77] MathurS, GlogowskaA, McAvoyE, RigholtC, RutherfordJ, WillingC, et al. (2014). Three-dimensional quantitative imaging of telomeres in buccal cells identifies mild, moderate, and severe Alzheimer's disease patients. J Alzheimers Dis, 39:35-48.24121960 10.3233/JAD-130866

[78] KhanTK, AlkonDL (2010). Early diagnostic accuracy and pathophysiologic relevance of an autopsy-confirmed Alzheimer's disease peripheral biomarker. Neurobiol Aging, 31:889-900.18760507 10.1016/j.neurobiolaging.2008.07.010

[79] FrancoisM, FenechMF, ThomasP, HorM, RembachA, MartinsRN, et al. (2016). High Content, Multi-Parameter Analyses in Buccal Cells to Identify Alzheimer's Disease. Curr Alzheimer Res, 13:787-799.26975368 10.2174/1567205013666160315112151

[80] JosephsKA, WeigandSD, WhitwellJL (2022). Characterizing Amyloid-Positive Individuals With Normal Tau PET Levels After 5 Years. Neurology, 98:e2282-e2292.35314506 10.1212/WNL.0000000000200287PMC9162162

[81] LeeYS, YounH, JeongHG, LeeTJ, HanJW, ParkJH, et al. (2021). Cost-effectiveness of using amyloid positron emission tomography in individuals with mild cognitive impairment. Cost Eff Resour Alloc, 19:50.34391439 10.1186/s12962-021-00300-9PMC8364075

[82] d'Abramo C, D'Adamio L, Giliberto L (2020). Significance of Blood and Cerebrospinal Fluid Biomarkers for Alzheimer's Disease: Sensitivity, Specificity and Potential for Clinical Use. J Pers Med, 10. 10.3390/jpm10030116 PMC7565390 32911755

[83] Mirzaie A, Nasrollahpour H, Khalilzadeh B, Jamali AA, Spiteri RJ, Yousefi H, et al. (2023). Cerebrospinal fluid: A specific biofluid for the biosensing of Alzheimer's diseases biomarkers. TrAC Trends in Analytical Chemistry, 166:117174.

[84] QuispialayaKM, TherriaultJ, AliagaA, ZimmermannM, Fernandez-AriasJ, LussierF, et al. (2022). Discordance and Concordance Between Cerebrospinal and [<sup>18</sup>F]FDG-PET Biomarkers in Assessing Atypical and Early-Onset AD Dementia Cases. Neurology, 99:e2428-e2436.36266044 10.1212/WNL.0000000000201198PMC9728035

[85] ChenYR, LiangCS, ChuH, VossJ, KangXL, O'ConnellG, et al. (2021). Diagnostic accuracy of blood biomarkers for Alzheimer's disease and amnestic mild cognitive impairment: A meta-analysis. Ageing Res Rev, 71:101446.34391944 10.1016/j.arr.2021.101446

[86] ParaskevaidiM, AllsopD, KarimS, MartinFL, CreanS (2020). Diagnostic Biomarkers for Alzheimer's Disease Using Non-Invasive Specimens. J Clin Med, 9.32492907 10.3390/jcm9061673PMC7356561

[87] RoostermanD, GoergeT, SchneiderSW, BunnettNW, SteinhoffM (2006). Neuronal control of skin function: the skin as a neuroimmunoendocrine organ. Physiol Rev, 86:1309-1379.17015491 10.1152/physrev.00026.2005

[88] WangJY, ZhangY, ChenY, WangY, LiSY, WangYF, et al. (2021). Mechanisms underlying antidepressant effect of transcutaneous auricular vagus nerve stimulation on CUMS model rats based on hippocampal alpha7nAchR/NF-kappaB signal pathway. J Neuroinflammation, 18:291.34920740 10.1186/s12974-021-02341-6PMC8680337

[89] Kaczmarczyk R, Tejera D, Simon BJ, Heneka MT (2017). Microglia modulation through external vagus nerve stimulation in a murine model of Alzheimer's disease. [J] Neurochem. 10.1111/jnc.14284 29266221

[90] YuY, JiangX, FangX, WangY, LiuP, LingJ, et al. (2023). Transauricular Vagal Nerve Stimulation at 40 Hz Inhibits Hippocampal P2X7R/NLRP3/Caspase-1 Signaling and Improves Spatial Learning and Memory in 6-Month-Old APP/PS1 Mice. Neuromodulation, 26:589-600.35595603 10.1016/j.neurom.2022.03.011

[91] Vargas-CaballeroM, WarmingH, WalkerR, HolmesC, CruickshankG, PatelB (2022). Vagus Nerve Stimulation as a Potential Therapy in Early Alzheimer's Disease: A Review. Front Hum Neurosci, 16:866434.35572001 10.3389/fnhum.2022.866434PMC9098960

[92] DolphinH, DyerAH, DukelowT, FinucaneC, ComminsS, KennellySP (2023). Safety and feasibility of transcutaneous vagus nerve stimulation in mild cognitive impairment: VINCI-AD study protocol. BMC Neurol, 23:289.37532979 10.1186/s12883-023-03320-5PMC10394887

[93] HansenN (2018). Memory Reinforcement and Attenuation by Activating the Human Locus Coeruleus via Transcutaneous Vagus Nerve Stimulation. Front Neurosci, 12:955.30686963 10.3389/fnins.2018.00955PMC6333671

[94] ZhouM, XuR, KaelberDC, GurneyME (2020). Tumor Necrosis Factor (TNF) blocking agents are associated with lower risk for Alzheimer's disease in patients with rheumatoid arthritis and psoriasis. PLoS One, 15:e0229819.32203525 10.1371/journal.pone.0229819PMC7089534

[95] DecourtB, LahiriDK, SabbaghMN (2017). Targeting Tumor Necrosis Factor Alpha for Alzheimer's Disease. Curr Alzheimer Res, 14:412-425.27697064 10.2174/1567205013666160930110551PMC5328927

[96] ChangR, YeeKL, SumbriaRK (2017). Tumor necrosis factor alpha Inhibition for Alzheimer's Disease. J Cent Nerv Syst Dis, 9:1179573517709278.28579870 10.1177/1179573517709278PMC5436834

[97] ShiJQ, ShenW, ChenJ, WangBR, ZhongLL, ZhuYW, et al. (2011). Anti-TNF-alpha reduces amyloid plaques and tau phosphorylation and induces CD11c-positive dendritic-like cell in the APP/PS1 transgenic mouse brains. Brain Res, 1368:239-247.20971085 10.1016/j.brainres.2010.10.053

[98] KimDH, ChoiSM, JhoJ, ParkMS, KangJ, ParkSJ, et al. (2016). Infliximab ameliorates AD-associated object recognition memory impairment. Behav Brain Res, 311:384-391.27265784 10.1016/j.bbr.2016.06.001

[99] AlkamT, NittaA, MizoguchiH, SaitoK, SeshimaM, ItohA, et al. (2008). Restraining tumor necrosis factor-alpha by thalidomide prevents the amyloid beta-induced impairment of recognition memory in mice. Behav Brain Res, 189:100-106.18325608 10.1016/j.bbr.2007.12.014

[100] Tobinick E, Gross H, Weinberger A, Cohen H (2006). TNF-alpha modulation for treatment of Alzheimer's disease: a 6-month pilot study. MedGenMed, 8:25. PMC1785182 16926764

[101] TobinickEL, GrossH (2008). Rapid cognitive improvement in Alzheimer's disease following perispinal etanercept administration. J Neuroinflammation, 5:2.18184433 10.1186/1742-2094-5-2PMC2211476

[102] ShiJQ, WangBR, JiangWW, ChenJ, ZhuYW, ZhongLL, et al. (2011). Cognitive improvement with intrathecal administration of infliximab in a woman with Alzheimer's disease. J Am Geriatr Soc, 59:1142-1144.21668921 10.1111/j.1532-5415.2011.03445.x

[103] ButchartJ, BrookL, HopkinsV, TeelingJ, PuntenerU, CullifordD, et al. (2015). Etanercept in Alzheimer disease: A randomized, placebo-controlled, double-blind, phase 2 trial. Neurology, 84:2161-2168.25934853 10.1212/WNL.0000000000001617PMC4451045

[104] ClausenBH, WirenfeldtM, HogedalSS, FrichLH, NielsenHH, SchroderHD, et al. (2020). Characterization of the TNF and IL-1 systems in human brain and blood after ischemic stroke. Acta Neuropathol Commun, 8:81.32503645 10.1186/s40478-020-00957-yPMC7273684

[105] ChangR, KnoxJ, ChangJ, DerbedrossianA, VasilevkoV, CribbsD, et al. (2017). Blood-Brain Barrier Penetrating Biologic TNF-alpha Inhibitor for Alzheimer's Disease. Mol Pharm, 14:2340-2349.28514851 10.1021/acs.molpharmaceut.7b00200

[106] LiY, FanH, NiM, ZhangW, FangF, SunJ, et al. (2022). Etanercept Reduces Neuron Injury and Neuroinflammation via Inactivating c-Jun N-terminal Kinase and Nuclear Factor-kappaB Pathways in Alzheimer's Disease: An In Vitro and In Vivo Investigation. Neuroscience, 484:140-150.35058089 10.1016/j.neuroscience.2021.11.001

[107] OuW, YangJ, SimanauskaiteJ, ChoiM, CastellanosDM, ChangR, et al. (2021). Biologic TNF-alpha inhibitors reduce microgliosis, neuronal loss, and tau phosphorylation in a transgenic mouse model of tauopathy. J Neuroinflammation, 18:312.34972522 10.1186/s12974-021-02332-7PMC8719395

[108] WuY, DuS, JohnsonJL, TungHY, LandersCT, LiuY, et al. (2019). Microglia and amyloid precursor protein coordinate control of transient Candida cerebritis with memory deficits. Nat Commun, 10:58.30610193 10.1038/s41467-018-07991-4PMC6320369

[109] AlonsoR, PisaD, AguadoB, CarrascoL (2017). Identification of Fungal Species in Brain Tissue from Alzheimer's Disease by Next-Generation Sequencing. J Alzheimers Dis, 58:55-67.28387676 10.3233/JAD-170058

[110] YeoIJ, YunJ, SonDJ, HanSB, HongJT (2020). Antifungal drug miconazole ameliorated memory deficits in a mouse model of LPS-induced memory loss through targeting iNOS. Cell Death Dis, 11:623.32796824 10.1038/s41419-020-2619-5PMC7429861

[111] BlockML, ZeccaL, HongJS (2007). Microglia-mediated neurotoxicity: uncovering the molecular mechanisms. Nat Rev Neurosci, 8:57-69.17180163 10.1038/nrn2038

[112] PalmerAA, StezoskiJP, Janesko-FeldmanK, KochanekPM, DrabekT (2022). Targeting TNFalpha-mediated cytotoxicity using thalidomide after experimental cardiac arrest in rats: An exploratory study. Exp Ther Med, 23:380.35495588 10.3892/etm.2022.11307PMC9019692

[113] TownerRA, GulejR, ZallesM, SaundersD, SmithN, LernerM, et al. (2021). Rapamycin restores brain vasculature, metabolism, and blood-brain barrier in an inflammaging model. Geroscience, 43:563-578.33846885 10.1007/s11357-021-00363-9PMC8110648

[114] WangG, LiZ, LiS, RenJ, SureshV, XuD, et al. (2019). Minocycline Preserves the Integrity and Permeability of BBB by Altering the Activity of DKK1-Wnt Signaling in ICH Model. Neuroscience, 415:135-146.31344398 10.1016/j.neuroscience.2019.06.038

[115] HuangD, WangQ, CaoY, YangH, LiM, WuF, et al. (2023). Multiscale NIR-II Imaging-Guided Brain-Targeted Drug Delivery Using Engineered Cell Membrane Nanoformulation for Alzheimer's Disease Therapy. ACS Nano, 17:5033-5046.36867454 10.1021/acsnano.2c12840

[116] Santos G, Pardi P (2021). ASSESSMENT OF BRAIN ESTERASE LEVELS IN PATIENTS WITH ALZHEIMER'S DISEASE.

[117] Pardi GAAdSaPC (2020). The Therapeutic Effects of Oral and Transdermal Rivastigmine for the Treatment of Alzheimer’s Disease. Journal of Pharmacy and Drug Development, 2:1-6.

[118] ChangCC, ChanL, ChouHH, YangYW, ChenTF, ChenTB, et al. (2021). Effectiveness of the 10 cm(2) Rivastigmine Patch in Taiwanese Patients with Mild-to-Moderate Alzheimer's Dementia: A 48-Week Real-World Observational Study. Adv Ther, 38:5286-5301.34506009 10.1007/s12325-021-01893-6PMC8478746

[119] WinbladB, CummingsJ, AndreasenN, GrossbergG, OnofrjM, SadowskyC, et al. (2007). A six-month double-blind, randomized, placebo-controlled study of a transdermal patch in Alzheimer's disease--rivastigmine patch versus capsule. Int J Geriatr Psychiatry, 22:456-467.17380489 10.1002/gps.1788

[120] BarthelH, GertzHJ, DreselS, PetersO, BartensteinP, BuergerK, et al. (2011). Cerebral amyloid-beta PET with florbetaben (18F) in patients with Alzheimer's disease and healthy controls: a multicentre phase 2 diagnostic study. Lancet Neurol, 10:424-435.21481640 10.1016/S1474-4422(11)70077-1

[121] WangL, BenzingerTL, SuY, ChristensenJ, FriedrichsenK, AldeaP, et al. (2016). Evaluation of Tau Imaging in Staging Alzheimer Disease and Revealing Interactions Between beta-Amyloid and Tauopathy. JAMA Neurol, 73:1070-1077.27454922 10.1001/jamaneurol.2016.2078PMC5237382

[122] ShojiM, MatsubaraE, KanaiM, WatanabeM, NakamuraT, TomidokoroY, et al. (1998). Combination assay of CSF tau, A beta 1-40 and A beta 1-42(43) as a biochemical marker of Alzheimer's disease. J Neurol Sci, 158:134-140.9702683 10.1016/s0022-510x(98)00122-1

[123] LewczukP, EsselmannH, OttoM, MalerJM, HenkelAW, HenkelMK, et al. (2004). Neurochemical diagnosis of Alzheimer's dementia by CSF Abeta42, Abeta42/Abeta40 ratio and total tau. Neurobiol Aging, 25:273-281.15123331 10.1016/S0197-4580(03)00086-1

[124] NutuM, ZetterbergH, LondosE, MinthonL, NaggaK, BlennowK, et al. (2013). Evaluation of the cerebrospinal fluid amyloid-beta1-42/amyloid-beta1-40 ratio measured by alpha-LISA to distinguish Alzheimer's disease from other dementia disorders. Dement Geriatr Cogn Disord, 36:99-110.23860354 10.1159/000353442

[125] StruyfsH, Van BroeckB, TimmersM, FransenE, SleegersK, Van BroeckhovenC, et al. (2015). Diagnostic Accuracy of Cerebrospinal Fluid Amyloid-beta Isoforms for Early and Differential Dementia Diagnosis. J Alzheimers Dis, 45:813-822.25633670 10.3233/JAD-141986

[126] ChirilaFV, XuG, FontaineD, KernG, KhanTK, BrandtJ, et al. (2022). Morphometric imaging biomarker identifies Alzheimer's disease even among mixed dementia patients. Sci Rep, 12:17675.36319674 10.1038/s41598-022-21796-yPMC9626495

[127] MocaliA, Della MalvaN, AbeteC, Mitidieri CostanzaVA, BavazzanoA, BoddiV, et al. (2014). Altered proteolysis in fibroblasts of Alzheimer patients with predictive implications for subjects at risk of disease. Int J Alzheimers Dis, 2014:520152.24949214 10.1155/2014/520152PMC4052202

[128] DetraitER, DanisB, LambertyY, FoerchP (2014). Peripheral administration of an anti-TNF-alpha receptor fusion protein counteracts the amyloid induced elevation of hippocampal TNF-alpha levels and memory deficits in mice. Neurochem Int, 72:10-13.24726770 10.1016/j.neuint.2014.04.001

[129] CaccamoA, MajumderS, RichardsonA, StrongR, OddoS (2010). Molecular interplay between mammalian target of rapamycin (mTOR), amyloid-beta, and Tau: effects on cognitive impairments. J Biol Chem, 285:13107-13120.20178983 10.1074/jbc.M110.100420PMC2857107

[130] SeabrookTJ, JiangL, MaierM, LemereCA (2006). Minocycline affects microglia activation, Abeta deposition, and behavior in APP-tg mice. Glia, 53:776-782.16534778 10.1002/glia.20338

[131] ChoiY, KimHS, ShinKY, KimEM, KimM, KimHS, et al. (2007). Minocycline attenuates neuronal cell death and improves cognitive impairment in Alzheimer's disease models. Neuropsychopharmacology, 32:2393-2404.17406652 10.1038/sj.npp.1301377

[132] FerrettiMT, AllardS, PartridgeV, DucatenzeilerA, CuelloAC (2012). Minocycline corrects early, pre-plaque neuroinflammation and inhibits BACE-1 in a transgenic model of Alzheimer's disease-like amyloid pathology. J Neuroinflammation, 9:62.22472085 10.1186/1742-2094-9-62PMC3352127

[133] HunterCL, QuinteroEM, GilstrapL, BhatNR, GranholmAC (2004). Minocycline protects basal forebrain cholinergic neurons from mu p75-saporin immunotoxic lesioning. Eur J Neurosci, 19:3305-3316.15217386 10.1111/j.0953-816X.2004.03439.x

[134] FanR, XuF, PrevitiML, DavisJ, GrandeAM, RobinsonJK, et al. (2007). Minocycline reduces microglial activation and improves behavioral deficits in a transgenic model of cerebral microvascular amyloid. J Neurosci, 27:3057-3063.17376966 10.1523/JNEUROSCI.4371-06.2007PMC6672462

[135] ParisD, GaneyNJ, LaporteV, PatelNS, Beaulieu-AbdelahadD, BachmeierC, et al. (2010). Reduction of beta-amyloid pathology by celastrol in a transgenic mouse model of Alzheimer's disease. J Neuroinflammation, 7:17.20211007 10.1186/1742-2094-7-17PMC2841120

[136] CramerPE, CirritoJR, WessonDW, LeeCY, KarloJC, ZinnAE, et al. (2012). ApoE-directed therapeutics rapidly clear beta-amyloid and reverse deficits in AD mouse models. Science, 335:1503-1506.22323736 10.1126/science.1217697PMC3651582

[137] YatawaraC, ZailanFZ, ChuaEV, LimLLH, SilvaE, WangJS, et al. (2021). The Efficacy of Transdermal Rivastigmine in Mild to Moderate Alzheimer's Disease with Concomitant Small Vessel Cerebrovascular Disease: Findings from an Open-Label Study. Clin Interv Aging, 16:301-309.33642856 10.2147/CIA.S290055PMC7903964

